# A Novel Trans-Impedance Matrix (TIM) Abnormality Pattern in Cochlear Implants

**DOI:** 10.3390/audiolres15020024

**Published:** 2025-03-02

**Authors:** Erica Pizzol, Sara Ghiselli, Patrizia Frontera, Daria Salsi, Domenico Cuda

**Affiliations:** 1 Department of Otolaryngology, AUSL Piacenza, 29121 Piacenza, Italy; s.ghiselli@ausl.pc.it (S.G.); p.frontera@ausl.pc.it (P.F.); d.salsi@ausl.pc.it (D.S.); domenicorosario.cuda@unipr.it (D.C.); 2 Department of Medicine and Surgery, University of Parma, 43125 Parma, Italy

**Keywords:** audiology, cochlear implant, hearing loss, trans-impedance matrix, internal part

## Abstract

In our clinical setting, we have identified a novel pattern of Trans-Impedance Matrix (TIM) measurement that we call ’scatter’, a measure characterised by a loss of definition in the heat and line maps. **Objective:** the aim of this study was to describe the basic characteristics of the anomaly pattern. The secondary purpose is to evaluate correlations between the “scatter” pattern and normal TIM by considering different parameters. **Methods:** the experimental sample, therefore, consisted of 565 patients (81.1% of people with a checked TIM at follow-up; M: 279, F: 286 and mean age: 27 years (sd 26). The scatter pattern was found in 55 devices (9.7%). We classified this pattern as severe (20 devices) or mild (35 devices) according to the visual extent of the abnormality. We considered the visual extension of the pattern, device lifetime, type of internal part of the CI, and auditory performance (speech audiometry in quiet at 65 dB and in noise—Ita Matrix Sentence Test). We also analysed two quantitive parameters: Shannon entropy and exponential decay. **Results:** a difference was found in these two quantitative parameters between the severe scatter, mild scatter, and normal TIM groups (*p*-value < 0.0001). The severe scatter group seems to be related to the type of device (CI24RE and CI512) and long device life (average 133 months); it did not show differences in audiology performances compared to the other groups. **Conclusions:** this result gives a numerical validation to the more subjective visual inspection approach. The scatter pattern is a novel, previously undescribed abnormality of TIM. It can vary from moderate to severe. A numerical basis to validate the inspection approach is described here.

## 1. Introduction

The correct insertion of the electrode cochlear implant array is dependent on several factors, including the geometry of the electrodes, the surgical approach, and the variability or abnormality of the cochlear anatomy. Implanting arrays can give rise to a number of issues that have the potential to affect patient outcomes. The most common reasons for revision surgery are partial or deep insertion of the array, folding of the electrode tip, kinking, or migration of the electrode into the scala vestibuli [1]. Proper electrode insertion during surgery is not always straightforward and requires careful evaluation [2,3]. The gold standard for assessing electrode positioning is a radiological examination, such as a CT scan. However, intraoperative examination is often limited by a lack of proper resources [4].

Objective measurements of the internal part of the cochlear implant are typically conducted during surgery. Techniques such as impedance analysis, neural response telemetry (NRT) [5], evoked stapedial reflex [6], and electrocochleography (ECochG) can be utilised. These measurements evaluate different aspects, e.g., NRT gives us information about the nerve action potential, impedance gives us information about the integrity of the electrodes and the environment of surrounding tissues, the evoked stapedial reflex is an objective measurement that can help us with the initial fitting of the cochlear implant, especially in patients with lipid disorders or in children, and, finally, EchoG is a peripheral electrophysiological analysis of the cochlea and the cochlear nerve. None of these, however, gives us an idea of the correct placement of the electrodes [7].

Technological advances have led to the incorporation of objective measures into clinical practice, including the checking of electrode position [8]. An objective method, trans-impedance matrix (TIM), has been developed over time. This technique is based on the impedance differences between the electrodes in the array. In a TIM assessment, the electrode potentials within the cochlea, from each electrode contact, are taken during monopolar stimulation of a single contact. This allows us to gain invaluable insight into the propagation of the electric field within the cochlea. The results are presented in a clear and concise manner, with both a heat map and a line graph based on the impedance value metric.

This method is well documented in the literature as a tool for confirming electrode placement and allows clear differentiation between correct and incorrect tip positions [9].

TIM has been used in postoperative follow-up to analyse and identify abnormalities and to differentiate between different types of aetiology [10]. In a previous study, we found that TIM maps discriminated between otosclerotic and non-syndromic genetic patients based on measurements of two parameters: Shannon entropy and exponential decay [10].

Shannon entropy (SE) can be defined as the “degree of disorder” in a system. Its increase is directly related to the increase in “disorder” of the system. Entropy, a scalar quantity in physics, determines the tendency of a system or body to exchange or transform energy during chemical or thermodynamic processes in a particular manner rather than others. It was assumed to provide information about the spread of the electric field in the cochlea and its associated degree of disorder.

The exponential decay (ED) parameter indicates the decrease in the current value of a physical quantity. This parameter was used to study the propagation of the electric field within the cochlea as represented by the trans-impedance values measured along the electrodes positioned at different distances from the source.

As part of our standard follow-up approach, we systematically obtain TIM maps where possible. Over time, we have noticed that some of the devices show a particular pattern that we have termed ‘Scatter’ (https://cdn.ymaws.com/www.acialliance.org/resource/resmgr/ci2023/ci2023_poster_abstracts.pdf Abstract number 142, performed on 7 October 2023). This is characterised by a loss of gradation and definition in the heat maps and a loss of linearity in the line graph.

The purpose of this paper is to describe the basic characteristics of these anomaly ‘Scatter’ patterns. The secondary purpose is to evaluate the correlations between the “Scatter” and the normal TIM by considering different parameters (quantitative parameters such as Shannon entropy and exponential decay, device lifetime, type of internal part of the CI, and auditory performance).

## 2. Materials and Methods

This retrospective, non-randomised observational study was conducted in a monocentric design at the outpatient clinic of the otolaryngology department of the “Guglielmo da Saliceto” Hospital in Piacenza, Italy.

This study was registered with the Ethics Committee of the Area Vasta Emilia Nord under number 191/2020/OSS/AUSLPC (date of approval 19/06/2020), and all participants signed the informed consent form during the scheduled CI follow-up.

As the TIM measure is manufactured by Cochlear, LTD, Sydney, Australia, only patients implanted with this brand were included in this study. TIM measurements are only possible in some CI models of the internal part. See the section below for model characteristics.

Subjects with malformations of the inner ear were excluded from this study because the malformation of the cochlea could result in abnormal electrode positioning, such as folded tips or partial insertion, or anomalies in telemetry responses of the cochlear implant. In addition, cases in which one or more electrodes were disconnected were excluded because of a limitation in the analysis software of the parameters of Shannon entropy and exponential decay, and cases in which there were positioning anomalies (also known in radiology), such as tip folding or kinking; this was performed to avoid the possibility that the parameters analysed could be affected by positioning anomalies.

### 2.1. Transimpedance Matrix (TIM)

The TIM measurement is an objective tool available through Cochlear®’s CustomSound EP, Macquaire University NSW 2019, Australia.

**Figure 1:**
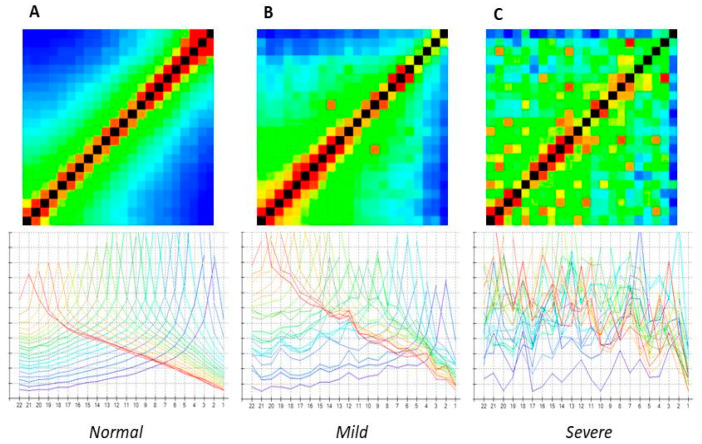
Heat map (**upper panels**) and line graph (**lower panels**) showing normal (**A**), mild (**B**), and severe (**C**) scatter patterns. In the figure, black color represent contacts of the stimulating electrodes. The red color indicates a high impedance and the blue color a low impedance.

The software generates a 22 × 22 matrix in a heat map format. The matrix is colour-coded from high (black and red) to low (green and blue) trans-impedance values. The highest values are recorded at the stimulating electrode contacts, which can be seen as a diagonal line from the lower left corner of the matrix (apical) to the upper right (basal), as shown in Figure 1 [11]. In addition to the heat maps, the results can also be displayed as line graphs. These are shown in the lower panels of Figure 1.

We have been acquiring TIM measurements during surgery since 2019. In addition, we have been retrospectively collecting data from previously operated patients during their routine follow-up.

### 2.2. Visual Inspection

Three audiologists visually inspected the TIM heat maps to identify and classify any ‘scatter’ pattern. They graded them into three categories: absent (‘normal’), mildly scattered (‘mild scatter’), and severely scattered (‘severe scatter’).

A ‘normal’ TIM was defined as having no visible alterations in both the line and heat map graphs. The ’mild scatter’ category was intended to represent a minimal but significant deviation from normal, while the ’severe scatter’ pattern was defined by an undeniable and widespread ’disorder’ in the heat map and line plots.

Paradigmatic examples are shown in Figure 1.

### 2.3. Derived Quantitative Analysis

#### 2.3.1. Subjects and Sampling

After excluding 73 patients implanted with models that do not support TIM measurements (CI24M; CI24R; Hybrid L24), 910 Cochlear^©^ recipients were initially screened for inclusion in this study. A significant proportion of them were followed over time with a TIM measurement (697 patients, or 76.6% of the eligible population). We then excluded 132 cases from further analysis according to the sampling criteria. They had one or more of the following characteristics: inner ear malformations, one or more electrodes off, and positioning abnormalities, such as tip fold-over or kinking. The final experimental sample thus consisted of 565 patients (81.1% of people with a checked TIM at follow-up). Patients were enrolled consecutively when they attended a routine clinical visit or surgical session. To avoid bias due to differences in the number of electrodes and different types of CI arrays, we preferred to analyse only the Cochlear brand.

#### 2.3.2. Transimpedance-Derived Quantitative Indexes

As indicated in our previous article by Vozzi et al. [10], the two indicators, Shannon entropy and exponential decay, were derived to validate the ’visual’ gradation model of the maps and to provide a numerical basis for more sophisticated analyses. We evaluated Shannon entropy as it is one of the indicators commonly used in the literature to describe information derived from images [12]. Instead, exponential decay was used to study the propagation of the electric field in the cochlea, as described by trans-impedance values measured along electrodes placed at different distances from the source. For a detailed explanation of how these indices were calculated, see Vozzi et al. [10]. The following devices were examined: CI24RE (*n* = 170), CI512 (*n* = 98), CI532 (*n* = 133), CI522 (*n* = 1), CI422 (*n* = 4), CI612 (*n* = 19), and CI632 (*n* = 140).

Device lifetime is defined as the period between CI surgery and the time of TIM measurement.

#### 2.3.3. Audiological Variables

This study evaluated three key metrics: Aided Pure Tone Average (PTA), Speech Perception Score (SPS) in quiet, and Signal-to-Noise Ratio (SNR). These tests are standardised and form part of the protocol for routine evaluations of patients followed in our centre. They are kept in the individual file. Measurements were taken on patients wearing only the CI undergoing TIM measurement.

The Pure Tone Average (PTA) is defined as the mean aided air warble tone threshold at frequencies of 500, 1000, 2000, and 4000 Hz.

The speech perception score (SPS) was measured in a quiet environment using pre-recorded lists of 20 disyllabic, phonetically balanced words [13] delivered at 65 dB HL by a loudspeaker at zero degrees of azimuth in a sound field. The patient was asked to repeat three lists, and the results were expressed as a percentage of correct perception out of a total of 60 words.

The signal-to-noise ratio (SNR) is a measure of hearing ability in noise. The Italian Matrix Sentence test [14] was used for evaluation. This is an adaptive test designed to determine the SNR (in dB) at which the subject can recognise 50% of the presented words. The evaluation begins with a training session. The patient is then given three randomised lists of 20 sentences. Each sentence comprises five semantically unpredictable words. The background noise is presented at a fixed level of 65 dB SPL, while the speech level is adaptively adjusted based on the subjects’ responses to achieve the SNR. The speakers were configured S0N0, with speech and noise coming from the same front speaker.

The audiological tests reported here refer to the aided condition with the implant switched on. In the case of bilateral implantation, two different data sets are acquired, one for the right and one for the left side, in accordance with the standard protocol at our centre. When necessary, as in the case of bimodal stimulation, the hearing aid was removed, and the ear was eventually masked with broadband noise. For simplicity, the data for each device are considered as a single case.

The audiological measurements were conducted using a Madsen^®^ Astera audiometer (Natus Medical Incorporated, Middleton, WI, USA). The frontal loudspeaker was positioned one metre away from the patients in a soundproof room.

### 2.4. Statistical Analysis

The analysis of entropy and exponential decay was conducted using MedCalc^®^ Statistical Software version 22.014 (MedCalc Software Ltd., Ostend, Belgium; https://www.medcalc.org; accessed on 7 November 2023). R Core Team (software), version 4.3.3, 2021 (https://www.R-project.org; accessed on 7 November 2023), was used for the analysis of device types, device lifetime, and audiological performance. The Kolmogorov–Smirnov test was used to verify the normality of the statistical distribution of the variables and to determine which variables were parametric. Tukey outlier detection was used to identify anomalous observations in the sample data. The Kruskal–Wallis test was used to confirm that the medians of different groups came from the same population (or from populations with the same median). The Kruskal–Wallis test (H-test) is an extension of the Wilcoxon test and is the correct test to use when testing the hypothesis that a number of unpaired samples originate from the same population. If the null hypothesis is rejected (*p* < 0.05), it can be concluded that there is a statistically significant difference between at least two of the subgroups. The Conover Post-hoc test was used for pairwise comparison of subgroups when the Kruskal–Wallis test was positive (i.e., *p* was less than the selected significance level) [15,16].

We reject the null hypothesis in the Kruskal–Wallis test when comparing device lifetime across grades. However, we do not reject it when comparing PTA, SPS, and SNR. The post-hoc analysis based on the Conover test (Benjamini and Hochberg *p*-value correction) indicates that the normal group is different from the other two.

## 3. Results

A ‘scatter’ pattern was identified in 55 out of 565 cases (9.7%). In 20 cases (36%), the scatter was graded as severe, while in the remaining 35 (63%) it was graded as mild. The distribution of ear side and sex was as follows: 44 right sides and 11 left sides; 25 females and 30 males.

The transimpedance pattern of the remaining 510 cases was classified as normal. The distribution of ear side and sex was as follows: 286 right sides and 224 left sides; 261 females and 249 males (see Table 1).

### 3.1. Shannon Entropy and Exponential Decay

**Figure 2:**
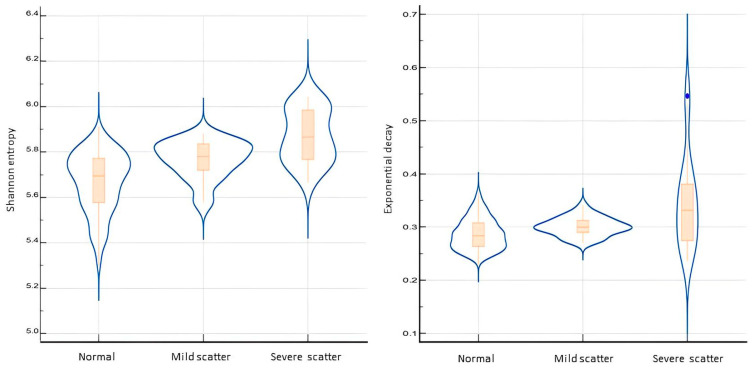
Shannon entropy (**left panel**) and exponential decay (**right panel**); both are plotted across different visual grades of transimpedance scattering.

The severe scatter TIM patterns unquestionably exhibit significantly higher mean values for the Shannon entropy and exponential decay parameters (5.87 and 0.47, respectively) compared to the mild (5.75 and 0.31) and normal (5.65 and 0.29) TIM patterns. The *p*-value for the comparison between the normal and mild groups is <0.0001, as well as the *p*-value for the comparison between the normal and severe groups is <0.0001. The *p*-value for the comparison between the mild and severe groups is 0.0054. The exponential decay comparisons demonstrated a *p*-value of 0.0009 between the normal and mild groups and a *p*-value of 0.0013 between the normal and severe groups. However, there was no significant difference in entropy between the mild and severe groups (*p*-value 0.085). See Figure 2.

### 3.2. Device Types

The scattering was only found on the transimpedance heat maps of three types of CI and exactly 34 (20%) in CI24RE; 16 (16.3%) in CI512 and 5 (3.7%) in CI532. CI612 and CI632 were actually not involved in the Scatter group. We found a higher frequency of Good TIM in the CI532 and CI632 models.

### 3.3. Device Lifetime

**Figure 3:**
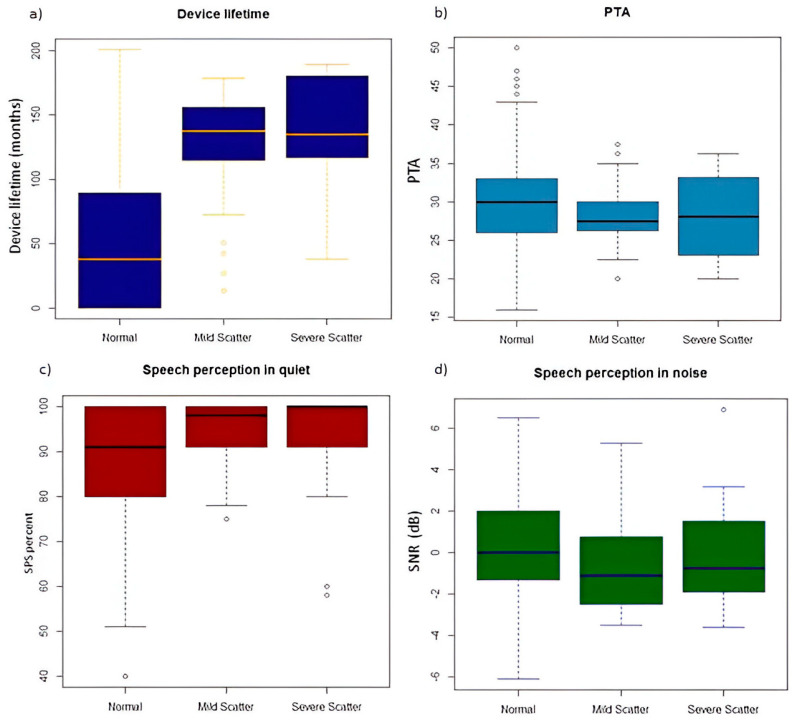
Distribution of device lifetime (**a**), aided pure tone threshold (**b**), speech perception score in quiet (**c**), and speech-to-noise ratio (**d**) across different transimpedance matrix patterns.

In the presence of scattering of any degree (mild or severe), the mean device lifetime was 133 months (sd 41.6; range 13–189) (Table 1 and Figure 3a).

In detail, the device lifetime was 129 months (sd 42.4; range 13–178) in the case of a mild scatter pattern and 139 months (sd 40.7; range 37–189) when a severe scatter pattern was found. Instead, devices with a normal TIM pattern (n 510) had a mean lifetime of 51 months (sd 50.9; range 0–201). The differences between devices with no scatter and those with mild and severe scatter were both highly significant in terms of TIM (*p*-value < 0.001), whereas the difference in device lifetime between mild and severe scatter was not significant (Figure 3a).

### 3.4. Audiological Performance

The aided pure-tone average threshold (PTA) was 30.1 dB HL (range 16–50) in the normal pattern group and 28.4 dB HL (range 20–38) in the scattered groups. In particular, it was 28 dB HL (range 20–38) in the mild group and 28.4 dB HL (range 20–36) in the severe scatter group. All of the reported values were similar and there were no statistically significant differences (Figure 3b).

The Speech Perception Score (SPS) in the quiet environment was 87.7% (range 40–100) in the normal pattern group and 94% (range 58–100) in the scattered groups. In particular, the SPS was 94.3% (range 75–100) in the mild scatter and 92.3% (range 58–100) in the severe scatter. All reported values were similar and there were no statistically significant differences (Figure 3c).

Speech perception in noise, reported as the speech-to-noise ratio (SNR), was 0.26 dB (range −6.1/+6.5) in the normal pattern, −0.71 dB (range −3.5/+5.3) in mild scatter, and 0.06 dB (range −3.6/+6.9) in severe scatter. These differences were not statistically significant (Figure 3d).

## 4. Discussion

To the best of our knowledge, this study is the first in the literature to describe a novel pattern of transimpedance matrix in cochlear implant recipients. We have termed this pattern ‘scatter’ due to the presence of a sparse inhomogeneity of colouration in the heat map. We categorised TIM scatter as mild or severe based on visual assessment. We identified a ‘scatter’ pattern in 55 out of 565 devices (9.7%). In 20 devices (36%), the scatter was graded as severe, while, in 35 devices (63%), it was graded as mild.

The visual classification was validated by a numerical analysis, which compared anomalies with the normal heat map. We calculated parameters related to system disorder (Shannon entropy) and the decrease in the current value of a physical quantity (exponential decay).

The Shannon entropy and exponential decay parameters were significantly higher in the severe scattering group than in the normal group and higher in the mild scattering group than in the normal. Less statistical differences were found between the two scatter groups. These data would be associated with greater disorder in the scatter system with a greater dispersion in the heat map and a lack of linearity in the line graph. The higher values of these two quantitative parameters proved sensitive in discriminating the groups with and without scatter patterns. Similar results were obtained in our previous study regarding TIM differences in congenital *vs.* otosclerotic CI users [10].

Based on the correlation between the “Scatter” and the normal TIM, we found significant results in the device lifetime and type of internal part parameters.

The average lifetime of devices with a scatter pattern was significantly longer than the normal TIM group (133 vs. 51 months). There was also a discernible trend between severe and mild scatter, with a mean use of 139 months in the severe and 129 months in the mild group.

TIM scatter was only observed with the CI24RE, CI512, and CI532 models, and never with the CI632, CI612, or CI622 models. This observation can be attributed to the differing device lifespans. Indeed, the CI24RE, CI512, and CI532 models were manufactured and utilised until 2018. Conversely, the CI632, CI612 or CI622 models have been manufactured and employed in clinics since 2019, and, therefore, the data collected with these models pertain to younger devices. It is, however, necessary to verify this hypothesis over time.

We can, therefore, state that the CI24RE, CI512, and CI532 models, having been used for the longest amount of time, have a longer device life and, consequently, the device lifetime and CI models are closely linked.

By comparing our results with those in the literature, we found that there are no studies on the time course of the impedance matrix (TIM). On the contrary, there is literature demonstrating a change in CI impedance parameters in the first months of CI use and a change in e-CAP parameters in the first years of CI use. After a long period of time, there is stability of these parameters [17,18].

The scatter TIM that we found in longer-term CI users can be linked to extended exposure to electrical stimulation and possible resulting tissue alterations.

A long duration of exposure to electrical stimulation can lead to a significant increase in both tissue response and platinum particles within the tissue capsule surrounding the electrode array [19]. High charge intensities or direct current can cause irreversible electrochemical reactions, including the hydrolysis of water, dissolution of platinum, and reduction of O_2_. These reactions can alter the electrolytic environment adjacent to the electrode, potentially causing tissue damage or electrode corrosion [20,21]. However, the long-term effects of electrical stimulation are not well understood. It is possible that even ‘safe’ stimulation, such as that used in modern systems, may cause slight corrosion effects on the electrodes or their silicone insulation [19,21]. These effects may ultimately be responsible for irregularities in trans-impedance measurements over years of device use.

For these reasons, long-term clinical use of these CI devices has highlighted the need for greater caution in terms of safety limits [19,20,21].

From a practical point of view, it is important to note that scattering, even in its severe form, does not appear to have a significant impact on clinical performance. There are no differences between normal and scattered devices in terms of the audibility of signals (PTA) or speech perception in a quiet environment (SPS). Also, no significant differences were found for listening in an environment containing noise. As scatter appears to be clearly related to the duration of stimulation, regular monitoring will be required to rule out the occurrence of subsequent declines in performance and also to evaluate other clinical parameters (for example, stability requirements or device calibration). At the same time, it will be necessary to ’map’ the transimpedance with other devices to determine whether it is model-specific or generalisable, especially as the appropriate software to detect it becomes available for clinical use with other brands. This phenomenon of TIM will need to be assessed over time, especially in cases where we have severe scatter conditions, to identify any future problems.

## 5. Conclusions

The scatter pattern is a novel, previously undescribed anomaly of the transimpedance matrix present in 9.7% of our large sample of cochlear implant recipients. It does not significantly impair speech perception. It seems to be related to the type of device and its long lifetime. To date, there is no reason to define the scatter fenome as a rupture or anomaly of the device, but we suggest paying attention to these patients. It is particularly important to perform serial follow-ups in patients with severe scatter TIM related to higher values of Shannon entropy and exponential decay values. More data and longer follow-ups are needed to fully understand the phenomenon and its impact on hearing performance over time.

## Figures and Tables

**Table 1:** Demographic and basic clinical variables of normal, mildly scattered, and severely scattered TIM groups.

Grade TIM	Patients (n)	Age at CI (Years)	Sex		Side of CI		Device Lifetime (Months)
			Female	Male	Right	Left	
Severe scatter	20	12 (sd 19.6)	11	9	17	3	139 (sd 40.7)
Mild scatter	35	14 (sd 19.8)	14	21	27	8	129 (sd 42.3)
Normal	510	29 (sd 26.3)	261	249	286	224	51 (sd 51)

## Data Availability

The data presented in this study are available on request from the corresponding author.
